# Toll-like Receptors and Celiac Disease

**DOI:** 10.3390/ijms24010265

**Published:** 2022-12-23

**Authors:** Diana Talipova, Aiganym Smagulova, Dimitri Poddighe

**Affiliations:** 1 Department of Medicine, Nazarbayev University School of Medicine, Astana 010000, Kazakhstan; 2 Clinical Academic Department of Pediatrics, National Research Center for Maternal and Child Health, University Medical Center, Astana 010000, Kazakhstan

**Keywords:** celiac disease, gluten, toll-like receptors (TLR), innate immunity, pattern recognition receptors (PRRs), pathogen-associated molecular patterns (PAMPs)

## Abstract

Celiac disease (CD) is an immune-mediated disorder triggered by dietary gluten intake in some genetically predisposed individuals; however, the additional non-HLA-related genetic factors implicated in CD immunopathogenesis are not well-defined. The role of the innate immune system in autoimmunity has emerged in the last few years. Genetic polymorphisms of some pattern-recognition receptors, including toll-like receptors (TLRs), have been associated with several autoimmune disorders. In this review, we summarize and discuss the evidence from basic research and clinical studies as regards the potential role of TLRs in CD immunopathogenesis. The evidence supporting the role of TLRs in CD immunopathogenesis is limited, especially in terms of basic research. However, differences in the expression and activation of TLRs between active CD patients from one side, and controls and treated CD patients from the other side, have been described in some clinical studies. Therefore, TLRs may be part of those non-HLA-related genetic factors implicated in CD etiopathogenesis, considering their potential role in the interaction between the host immune system and some environmental factors (including viral infections and gut microbiota), which are included in the list of candidate agents potentially contributing to the determination of CD risk in genetically predisposed individuals exposed to dietary gluten intake. Further basic research and clinical studies focused on TLRs in the context of CD and other gluten-related disorders are needed.

## 1. Introduction

Pattern recognition receptors (PRRs) are a family of germline-encoded molecules capable of detecting the presence of microbes and damaged host cells. Indeed, PRRs are considered fundamental components of the innate immune system since they serve as “sensors” alerting for potential threats and stimulating the inflammatory response before the antigen-specific mechanisms of the adaptive immune response can be activated [1].

Overall, PRRs can sense a wide range of pathogen-specific molecules, collectively indicated as pathogen-associated molecular patterns (PAMPs), which are basically core molecular motifs expressed by various pathogens, such as bacterial/fungal cell wall components and viral nucleic acids. Additionally, PRRs are also able to recognize different molecules produced by damaged or stressed cells, which are named damage-associated molecular patterns (DAMPs) [2,3].

To date, several classes of PRRs have been identified. First of all, they are classified according to their cellular localization. Membrane-bound PRRs include toll-like receptors (TLRs) and C-type lectin receptors (CTLs). Cytoplasmic PRRs include nucleotide-binding oligomerization domain (NOD)-like receptor (NLRs), retinoic acid-inducible gene (RIG)-I-like receptors (RLRs) and are absent in melanoma 2 (AIM2)-like receptors (ALRs) [4,5,6]. Notably, many soluble molecules can also work as PRRs, including complement receptors, collectins (such as mannan-binding lectin, MBL), ficolins, pentraxins (such as serum amyloid and C-reactive protein), and several others [7].

In addition to protecting against infections in general, PRRs have been implicated in the pathogenesis of autoimmune and immune-mediated disorders [8]. For instance, cumulative data indicated that NLRs may play a role in inflammatory bowel diseases, rheumatoid arthritis, systemic lupus erythematosus, psoriasis, multiple sclerosis, and other autoimmune disorders. [9] Similarly, several studies have suggested that some genetic variants of TLRs could also contribute to the pathogenesis of type 1 diabetes mellitus, Graves’ disease, rheumatoid arthritis, systemic lupus erythematosus, and multiple sclerosis [10].

Celiac disease (CD) is an immune-mediated disorder triggered by gluten intake. The primary target of CD is the gut: indeed, by definition, CD is diagnosed in the presence of an enteropathy characterized by intraepithelial lymphocyte infiltration and variable degrees of villous mucosal atrophy of the small intestine, which can be successfully treated by implementing a strict gluten-free diet (GFD). [11] However, CD patients often show variable extra-intestinal manifestations since numerous organs can be affected by the immunopathological process [12,13]. Notably, a specific genetic HLA-DQ background is required to develop CD: indeed, almost all CD patients basically possess one or more allelic variants related to HLA-DQ2 and/or HLA-DQ8 heterodimers, which is a necessary, but not sufficient, factor to develop CD upon gluten dietary exposure [14,15]. Therefore, additional genetic, epigenetic, immunological, and environmental factors are likely to be implicated in the pathogenesis of CD at the individual level, along with the mandatory HLA predisposition and gluten intake [16,17,18].

Among these additional genetic and immunological factors, TLRs may also play a role in the immunopathogenesis of CD. In this review, we summarize and discuss the evidence from basic research and clinical studies on this matter.

## 2. Toll-like Receptors (TLRs)

Toll-like receptors (TLRs) are type I transmembrane proteins expressed by many innate immune cells, including dendritic cells, monocytes, macrophages; however, these molecules have been also detected on B cells and specific types of T cells [8,19,20].

TLRs are integral transmembrane glycoproteins containing a leucine-rich repeats (LRRs) domain and a toll/interleukin (IL)-1 receptor (TIR) domain; between the N-terminal part of the LRRs domain and the C-terminal of the TIR domain, there is a single-spanning transmembrane domain, which is homologous to IL-1R family proteins and is important to keep the receptor in the correct orientation through the cell membrane [20,21]. Indeed, the LRRs domain is oriented toward the “extracellular” side and contains multiple (7–19) leucine (L)-rich sequences (or repeats), which overall harbor 24–29 amino acids. In this LRRs domain, two main types of repeats were identified: “typical” (T) motifs (LxxLxLxxNxLxxLxxxxF/LxxLxx), and “bacterial” (S) motifs (LxxLxLxxNx LxxLPx(x) LPxx). This LRRs domain is supposed to form a horseshoe structure, which can accommodate and, thus, recognize a variety of PAMPs. Thus, it is involved in a diverse set of TLR ligand–receptor interactions and signal transduction [20,22]. The internally oriented TIR domain usually consists of approximately 150 amino acid residues; it forms a kind of intracellular tail, which activates the downstream signaling inside the cell [22,23].

TLRs are initially synthesized in the endoplasmic reticulum; then, passing through the Golgi complex, they are directed either to the cell membrane, or to the endosomal compartments to be included in their membrane. The transportation of “intracellular TLRs” to endosomes is controlled by Unc-93 homolog B1 protein (UNC93B1), whereas the trafficking of “extra-cellular” TLRs from the endoplasmic reticulum to the plasma membrane requires other proteins, including PRAT4A and gp96 [24,25].

To date, 10 human TLRs have been identified [25,26]. As already mentioned, TLRs can be subdivided into two groups based on their primary localization, either plasma membrane or endosomes. Notably, the former ones (“extracellular” TLRs) are mainly activated by lipids or proteins contained in the microbial membranes, whereas the latter ones (“intra-cellular” TLRs) mainly recognizes microbial nucleic acids. In humans, TLR1, TLR2, TLR4, TLR5, TLR6 and TLR10 are largely expressed on the cell surface; TLR3, TLR7, TLR8, and TLR9 are included in endosomal membranes [24,27].

The PAMPs recognition and binding by TLRs induces the expression of pro-inflammatory cytokines via two main pathways: the myeloid differentiation factor 88 (MyD88)-dependent signaling pathway and the (MyD88-independent) TIR domain-containing adaptor-inducing IFN-β (TRIF) pathway [28,29]. All TLRs, except for TLR3, rely on the MyD88 adaptor protein for the full activation of the intra-cellular signaling, and some of them (such as TLR7 and TLR9) are entirely and strictly dependent on MyD88. TLR4 is the only TLR that can use both MyD88-dependent and MyD88-independent (TRIF-dependent) signaling pathways [8,20,21,26].

Upon PAMPs-related TLRs activation, cytosolic TIR domain-containing adaptors (including MyD88, TRIF, TIR domain-containing adaptor protein -TIRAP-, TRIF-related adaptor molecule -TRAM-) are recruited [29,30]. These cytosolic adaptor proteins can finally activate several transcription factors (including NF-kB, IRFs and AP-1) through IRAKs, TRAF6, TAK1, and IKK complex. The final result is the variable expression of inflammatory cytokines, type I interferons, and INF-β [30,31].

For instance, MyD88 contains an N-terminal death domain (DD), which interacts with IL-1R-associated kinase-4 (IRAK-4) through its own DD. IRAK4 phosphorylates IRAK1 and IRAK2; they in turn activate TNF receptor-associated factor 6 (TRAF6); and TRAF6 also activates the MAPKs and/or TGF-β activated kinase 1 (TAK1). TAK1 phosphorylates IKKb. The IKK proteins phosphorylate IkBs, resulting in their degradation and the resultant nuclear translocation of NF-kB [29,32].

TRIF associates with TRAF3 and TRAF6, as well as receptor-interacting proteins 1 and 3 (RIP1 and RIP3). TRAF6 and RIP1, with the help of TRADD and TAK1, activate NF-kB and MAPKs to induce proinflammatory cytokines. TRAF3 links TBK1 to the TRIF-dependent pathway, which in combination with IKK3, phosphorylates and activates IRF3, leading to IFN-β production. TRIF strictly signals from an intracellular location, which results in the activation of type I interferons (IFN)s, whereas MyD88 can signal both when associated with the plasma membrane and from within endosomal compartments [33,34].

Overall, TLRs trigger signaling cascades that induce the generation of pro-inflammatory cytokines, such as IL-1, IL-6 and TNF-α, and type I interferons (IFNs) [30]. Released cytokines mediate the induction of additional inflammatory cytokines and the enzymes that synthesize other inflammatory mediators. For instance, cyclooxygenase-2 and type II phospholipase A2 are enzymes which are triggered by IL-1 and activate the prostaglandin production cascade. Interferons also initiate a signaling cascade, which may finally lead to the induction of more than 300 IFN-stimulated genes [31,35,36].

Table 1 schematically summarizes the main characteristics of each TLR in terms of cellular expression, ligands (and potential microbiological target), intra-cellular signaling, and cytokine response [8,20,21,24,25,26,36,37,38].

## 3. Interaction between Toll-like Receptors and Gluten: Pre-Clinical Evidence

### 3.1. Ex Vivo/In Vitro Cellular Experiments

Table 2 summarizes the available articles exploring the interaction between gluten components and TLRs by using different types of cellular systems [39,40,41,42,43,44].

According to our literature search, Nikulina et al. first tested “the hypothesis that wheat gluten is not only a target of adaptive immunity, but also modulates the function of APC [antigen-presenting cells]”. Overall, they suggested that wheat gluten could lower the threshold for immune responses by promoting the maturation of APCs, although it did not stimulate the production of classic proinflammatory mediators (TNF-α or MIP-1α). Among their set of experiments, these authors investigated the effect of gluten on the maturation of bone marrow-derived dendritic cells from C3H/HeJ mice, which express a mutated and LPS-hyporesponsive TLR4; however, their results indicated that TLR4 does not seem to be substantially involved in the gluten-induced maturation of bone marrow-derived dendritic cells [39].

Thomas et al. further investigated the interaction of gliadin peptides with the innate immune system: in detail, they used cellular systems with macrophages derived from some murine experimental models (including TLR4 and MyD88 knockout mice) and also used Chinese hamster ovary (CHO) reporter cell lines, some of which were transfected to express endogenous hamster TLR4 or human TLR2. In summary, they concluded that “the interaction of gliadin with macrophages elicits a MyD88-dependent pro-inflammatory cytokine milieu that facilitates the interaction of T cells with APCs, leading ultimately to the antigen-specific adaptive immune response seen in patients with CD”. Moreover, they suggested that such an interaction (and the related cytokines production) was dependent on TLR/IL-1R signaling pathways (which relies on MyD88 signaling) but was neither TLR2- nor TLR4-dependent [40].

Ciccocioppo et al. investigated the effect of gliadin stimulation on bone marrow-derived dendritic cells from HLA-DQ8 transgenic mice (expressing the human HLA-DQ8 molecule in the absence of endogenous MHC class II genes) and BALB/c mice. As specifically regards TLRs, these authors showed that α-chymotrypsin-treated gliadin can induce a significant upregulation of TLR4, TLR7 and TLR8 expression in dendritic cells from the HLA-DQ8 mouse, while this response was evident in BALB/c dendritic cells only for TLR8. No TLR9 response was detected in dendritic cells from either murine model. Notably, this study showed that digested gliadin was not able to induce the activation of dendritic cells, although it could induce their maturation. Then, these experiments suggested that gliadin could promote a TLRs response in dendritic cells expressing HLA-DQ8 molecules, which resulted to be also associated with an increased secretion of IFN-α. However, it is unclear whether the increased expression of TLRs was directly involved in the enhanced production of IFN-α and, thus, contributing to the pro-inflammatory milieu that may be implicated in CD immunopathogenesis [41].

Palova-Jelinkova et al. observed a remarkable caspase-1-dependent secretion of IL-1β and IL-1α (in addition to a mild production of IL-18) from peripheral blood mononuclear cells (PBMCs) and, in detail, monocytes from CD patients upon ex vivo stimulation with pepsin digest of wheat gliadin fraction (PDWGF). Then, in order to investigate the molecular basis of this finding, they took advantage of cell systems derived from some murine experimental models. Briefly, their experiments with murine (TLR2, TLR4 and TLR2/4 knock-out mice) bone-marrow-derived dendritic cells (BMDCs) stimulated with PDWGF showed that IL-1β production was strongly dependent on MyD88 and, to certain extent, on TRIF, which suggested that components of this gliadin digested fraction could induce signaling via TLR2 and TLR4 receptors [42].

A recent study by Herrera et al. investigated the role of 33-mer gliadin peptides and their aggregates in the activation of TLRs in murine macrophages. In addition to showing that 33-mer gliadin peptides oligomerize to form square-like and rod-like nanostructures, these authors concluded that 33-mer peptide can activate the innate immune system and induce the production TNF-α and other pro-inflammatory cytokines, such as IP-10/CXCL10. Notably, larger 33-mer supramolecular structures resulted to induce an overexpression of NF-κB via TLR2/TLR4-dependent pathways. This finding would support the hypothesis that gliadin peptides may directly activate TLRs-mediated mechanisms of innate inflammation as well [43].

The effect of the gliadin peptide p31–43 was the object of the recent study by Nanayakkara et al. In addition to showing that the IFN-α pathway was activated by this gliadin peptide in the duodenal mucosa of CD patients, these authors investigated its effects on CaCo-2 cells, an intestinal epithelial cell line which is responsive to gliadin: briefly, they showed that p31–43 gliadin peptide can induce an IFN-α-mediated innate immune response in CaCo-2 cells by activating the TLR7 pathway, as done by some viral agents [44]. Indeed, TLR7 is an endosomal receptor that specifically recognizes viral mRNAs and signals by forming a complex with MyD88; then, such an activated complex recruits the MAPKs and leads to NF-κB activation, in order to ultimately induce the expression of IFN-α and other factors promoting viral resistance [45].

To conclude, we should also mention a series of experiments investigating the effect of non-gluten components of wheat on TLRs. Junker et al. showed that α-amylase/trypsin inhibitors (ATIs) can activate a TLR4-dependent signaling and elicit a strong innate immune response [46]. These ATIs are a group of (non-gliadin related) proteins contained in the seeds of all cereals (including wheat, barley, rye, maize, millet, and rice) and, in detail, in the water soluble (albumin) fraction of wheat, of which they represent the most abundant protein component. [47] This TLR4-mediated promotion of intestinal inflammation by wheat ATIs activating gut and mesenteric lymph node myeloid cells, was also supported by the study by Zevallos et al. [48]. More recently, another research group also demonstrated that ATIs could stimulate primary human macrophages through the TLR4/NF-κB pathway [49,50].

Although the number of these studies is limited, and different cellular systems with variable gliadin stimulation were used, overall, these experiments seem to suggest that gliadin peptides may affect the production of some pro-inflammatory cytokines by innate immune cells, and this gliadin-induced response may also pass through TLRs-dependent pathways to some extent. Moreover, even non-gliadin components of wheat may affect innate immune cells through TLRs activation and, thus, potentially promote some events that may be somehow implicated in CD immunopathogenesis, too.

### 3.2. In Vivo Animal Studies

Table 3 summarizes the available articles exploring the role of TLRs on the immune-mediated intestinal disease in murine experimental models challenged with gluten or its components [51,52,53,54].

Silva et al. analyzed gliadin sensitization and oral challenge in HCD4/HLA-DQ8 transgenic mice, which are characterized by HLA-DQ8-related mild intraepithelial lymphocytosis, gluten-dependent intestinal barrier dysfunction, even if that occurs in the absence of mucosal atrophy. These authors observed some changes in mucosal dendritic cells (DCs) markers distribution (CD103 and CD11c) in gliadin-treated mice: notably, these changes in DCs markers were not associated with the patterns of TLR2 or TLR4 cellular expression [51].

Araya et al. investigated whether gliadin peptide p31–43 elicits innate immune activation in murine small intestine. Although the effects of this gliadin peptide were dependent on MyD88, they were not associated with the activation of TLR4: indeed, treatment with gliadin peptide p31–43 in TLR4-deficient mice induced similar morphological changes as in wild-type mice. As an ancillary finding, these authors reported that the inflammatory changes induced by gliadin peptide p31–43 were enhanced by the co-administration of the TLR3 agonist polyinosinic:polycytidylic acid (poly I:C), which is a synthetic analog of dsRNA that mimics an innate response to viral infection [52,54]. This contribution of TLR3 expressed on innate lymphoid cells upon poly I:C stimulation for promoting the production of inflammatory mediators (in detail, TNF-α) in a mouse model of intestinal atrophy, was also reported by Marafini et al. [53].

Therefore, one might speculate that TLRs may be also involved in CD pathogenesis as mediators of environmental co-factors such as viral infections, which are considered as potential candidates and additional factors involved in CD etiology at the individual level. However, that could be a general mechanism of inflammatory exacerbation induced by viral infections in the context of any potential immune-mediated dietary intolerance, according to one of the aforementioned studies by Araya et al., who specifically and previously investigated this mechanism of poly I:C-induced enteropathy in gliadin-sensitive NOD-DQ8 mice [52].

Moreover, the article by Junker et al. mentioned in the previous subsection, in addition to analyzing the response of macrophages, monocytes, and dendritic cells to ATIs, reported that TLR-4 deficient mice were protected from intestinal and systemic immune responses upon oral challenge with ATIs [46]. Therefore, TLR4 may be indirectly implicated in the promotion of an inflammatory gut environment upon stimulation by ATIs, which are contained in wheat along with gluten.

Despite such a very small number of animal studies investigating the TLR-mediated effect of gluten components on murine models of intestinal atrophy, they seem to suggest a potential role of TLR2 and TLR4 in such a pathological setting.

## 4. Toll-like Receptors, Gluten and Celiac Disease: Clinical Evidence

All the available clinical studies investigating TLRs implication in CD patients specifically, are summarized in Table 4 [53,55,56,57,58,59,60,61,62,63,64,65,66,67,68,69].

The first clinical research on this specific topic dated back to 2006, when Zanoni et al. published a case-control study reporting that a subset of anti-transglutaminase IgA antibodies could bind the rotavirus-derived protein VP-7 as well as other self-antigens, including desmoglein 1 and TLR-4. These authors suggested that such an autoantibody detectable in active CD patients (but not in those on GFD) may bind TLR4 on monocytes and increase the expression of activation molecules (such as CD83 and CD40) and the production of pro-inflammatory mediators [55].

Eventually, several clinical studies focused on the potential contribution of TLR4 in CD immunopathogenesis. Santin et al. analyzed two coding single nucleotide polymorphisms (SNPs) of TLR4 (Asp299Gly and Thr399Ile) in a case-control study: no differences in allelic distribution between CD patients and controls was observed [57]. In their study analyzing the TLR4 Aþ896G SNP, Dezsofi et al. did not find any differences between CD and control groups either [58]. Fernandez-Jimenez et al. performed a copy number analysis of TLR4 gene, which did not show any variation between CD patients and controls. These authors also investigated TLR2 gene copy number with similar negative findings as described for TLR4 [60]. Notably, no specific TLR2 polymorphisms analyses were published. However, very recently, Cerqueira et al. suggested that the TLR7/TLR8 region (and, in detail, Rs5979785 SNP) was associated with CD onset before 7 years of age in girls; however, the mechanistic significance of this finding is unclear, since a previous analysis did not identify any regulatory impact of Rs5979785 on the TLR7/TLR8 transcription. Overall, this genome-wide association study has not identified any TLRs polymorphisms as clearly associated to CD susceptibility [69].

Most gene expression studies concomitantly investigated both TLR2 and TLR4. Szebeni et al. analyzed TLR2 and TLR4 mRNA expression in the duodenal mucosa of children with treated CD, untreated CD, and controls. TLR2 and TLR4 mRNA and also protein expression was higher in CD patients; interestingly, such an increased mRNA expression was more remarkable in the duodenal mucosa of CD children on GFD than in untreated CD patients. Moreover, these authors also assessed TLR3 mRNA expression, which resulted to be increased in the duodenal mucosa of treated CD children compared with untreated CD and controls, whereas TLR3 protein was only detected in the duodenal mucosa of treated CD patients. This research was the first clinical study clearly suggesting a potential alteration of TLRs expression in CD patients [56].

Increased TLR4 mRNA expression was also demonstrated in the peripheral blood monocytes of untreated CD children by Brynychova et al., whereas no changes were detected as regards TLR2 mRNA expression. Additionally, these authors also investigated TLR7 mRNA expression in monocytes, which resulted in being significantly increased in newly diagnosed CD children, compared to both controls and treated CD patients [65]. Cseh et al. evaluated TLR2 and TLR4 expression on peripheral blood leukocytes by flow cytometry, in the context of a more general immunophenotyping study in CD children and controls diagnosed with functional abdominal pain. In addition to assessing specific surface markers on lymphocytes and innate immune cells, these authors also analyzed TLR2 and TLR4 expressed by dendritic cells and monocytes. First, they reported that dendritic cells (particularly, myeloid dendritic cells) were more prevalent in CD than in controls; moreover, they also reported an increased expression of both TLR2 and TLR4 on these cells. Notably, TLR4 expression on these antigen-presenting cells normalized after GFD, unlike TLR2 [59]. Kumar et al. specifically assessed TLR2 expression by flow cytometry in T regulatory lymphocytes [66].

Indeed, TLRs expression was previously described on T cells and, in detail, TLR2 was suggested as being implicated in controlling the expansion and function of regulatory T cells [70,71]. Increased expression of TLR2 was detected on induced T regulatory cells obtained from CD patients compared to controls, and a milder, but still significant, difference was maintained in patients on GFD, too. Conversely, no difference in the expression of TLR2 on natural T regulatory cells was observed among these three study groups [66].

Westerholm-Ormio et al. analyzed TLR2 and TLR4 cell expression by immunohistochemistry in the duodenal mucosa of pediatric patients affected with food allergy, including 10 CD children. The density of TLR4 positive cells in the lamina propria was significantly higher in CD patients than in controls; a similar, but much milder and not significant, trend was also observed for TLR2 positive cells. Interestingly, they also reported a positive correlation between the densities of cells harboring TLR2 and TLR4, and the densities of TCRδγ+ and Foxp3+ cells. Moreover, they also analyzed the mRNA expression in these duodenal biopsies: TLR2 mRNA expression was significantly reduced in the small intestinal mucosa of CD patients compared with controls, whereas TLR4 mRNA expression did not differ significantly [61].

Kalliomaki et al. more extensively assessed TLR gene expression in the intestinal mucosa of CD children (both treated and untreated) and controls. In detail, they analyzed TLR2, TLR3, TLR4, TLR5, TLR9, and also a regulator of TLRs, so-called Toll-interacting protein (TOLLIP). Briefly, in statistical terms, the main significant findings were a reduction in TLR2 mRNA expression in untreated and treated CD patients compared to controls, whereas TLR9 mRNA expression was increased in untreated CD patients only, who also showed a downregulation of TOLLIP mRNA expression. Notably, these authors concomitantly performed a microbiota analysis in order to assess the total bacterial number and 10 bacterial groups/species: overall, they did not find any significant differences in the amounts or frequencies of these bacteria between the study groups. However, they did not make any specific correlation analysis with the aforementioned immunological findings [62]. In the paper by Cheng et al., the same research group also highlighted that the increased TLR2 expression was positively correlated with the expression of tight junction protein ZO-1; moreover, they hypothesized that an increased TLR9 expression and signaling in the duodenum might contribute to the Th1 response (and increased IFN-γ) observed in the small intestinal mucosa of CD patients, persisting even during GFD regimen [64].

Another TLRs expression study was published by Eiro et al., who assessed TLR3, TLR4, and TLR7 (in addition to some interleukins and transcription factors) mRNA and protein expression in the duodenal biopsies from CD children and adults. As regards TLRs analysis, the only significant result was the higher protein expression of TLR4 in CD patients compared to controls; notably, there were no significant differences between pediatric and adult CD samples [63]. In their study investigating the role of innate lymphoid cells in CD, Marafini et al. also examined TLRs expression on these cells obtained from the duodenal mucosa of CD patients. In detail, immunostaining for TLR2, TLR3, TLR4, TLR7 and TLR9 was used. In active CD patients, approximately 10% of innate lymphoid cells expressed TLR2, 26% expressed TLR3 and 20% expressed TLR9 with no significant difference between CD patients (active and inactive) and controls. No relevant TLR4 and TLR7 expression was detected on these cells [53].

Recently, Ghasiyari et al. published a large case-control study (120 CD patients and 120 controls) where mRNA expression of several TLRs (TLR2, TLR4, TLR7, and TLR9) was assessed in the peripheral blood for all patients and in the intestinal mucosa for 20 randomly selected samples only. TLR4 and TLR9 mRNA expression was significantly increased in blood of CD patients compared to controls, whereas TLR2 and TLR7 mRNA expression was not statistically different between these groups. As regards duodenal mucosa specimens, TLR2 and TLR4 mRNA expression was increased in CD patients compared to controls, whereas TLR9 mRNA expression was significantly decreased in CD patients; no significant difference in the expression of TLR7 mRNA was observed between the study groups [67].

Additional research tried to investigate some functional aspects of TLR signaling in CD patients. Van Leeuwen et al. investigated IL-21 and IL-17A expression and producing cells in duodenal mucosa obtained from CD children: they demonstrated that increased IL-21 production was present in pediatric CD patients, mostly by CD4+ cells, which also secreted IFN-γ and low levels of IL-10. Additionally, they assessed TLRs (2,3,4, and 7) expression on purified T cells from healthy donors (not CD patients) who were not better defined: briefly, this study showed that the co-stimulation with TLR3 ligands during poly-clonal T-cell activation significantly increased IL-21 secretion from CD4+ T cells, whereas TLR2 ligands selectively enhanced IL-17A. Therefore, this research may further support the functional role of TLRs in modulating the secretion of cytokines implicated in CD immunopathogenesis [72]. Finally, Brynychova et al. analyzed cell-free DNA in CD patients and controls. Cell-free DNA (cfDNA) modified by apoptotic processes is able to activate TLR9; however, telomeric sequences in this cfDNA pool may conversely inhibit this receptor. Although no quantitative or qualitative differences were detected between these groups, these authors documented the ability of cfDNA contained in CD plasma samples to stimulate the production of TLR9 mRNA, which were significantly reduced after cell-free DNA removal from the plasma samples of CD patients. Conversely, such an effect was not observed in the controls [68].

## 5. Conclusions

The evidence supporting the role of TLRs in CD immunopathogenesis is limited, especially in terms of basic research. However, studies based on cellular system experiments suggested that gliadin peptides may affect the activation of some innate immune cells and/or the production of related pro-inflammatory cytokines, also through TLRs-dependent pathways to some extent. In this regard, TLR2 and TLR4 resulted to be the most investigated innate immunity receptors. Evidence from animal (murine) experimental models is even more limited, unfortunately.

As regards clinical studies, differences in the TLRs expression and related innate cells activation between active CD patients from one side, and controls and treated CD patients from the other side, have been described in some articles. Even though no specific TLRs genes polymorphisms have been associated to CD risk, altered TLRs expression (especially for TLR2 and TLR4, but others as well) has been variably reported in the duodenal mucosal and/or peripheral blood leukocytes from CD patients. However, the heterogeneity of the study designs, methods and objectives does not allow to draw any final conclusion on the specific role of TLRs in CD immunopathogenesis, although their contribution can be hypothesized.

**Figure 1:**
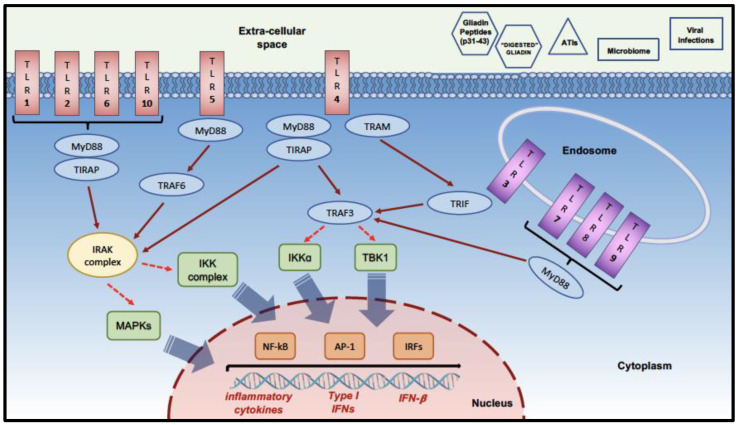
Graphical summary of the TLRs localization/main signaling pathways and potential factors affecting TLRs activation in Celiac Disease. **Abbreviations**: MyD88, myeloid differentiation primary response 88; TIRAP, toll-interleukin 1 receptor (TIR) domain containing adaptor Protein; TRAF, tumor necrosis factor receptor (TNFR)-associated factors; TRIF, TIR-domain-containing adapter-inducing interferon-β; MAPKs, mitogen-activated protein kinases; IKK, IkappaB kinase; TBK, TANK (TRAF family member-associated NF-kappa-B activator)-binding kinase; NF-kB, nuclear factor kappa-light-chain-enhancer of activated B cells; AP-1, activator protein 1; IRFs, interferon regulatory factors; ATIs, α-amylase/trypsin inhibitors.

Indeed, as graphically summarized in Figure 1, TLRs may be part of those non-HLA-related genetic factors which could be implicated in CD etiopathogenesis, considering their potential role in the interaction between the host immune system and some environmental factors, such as gliadin-digested peptides, other non-gliadin related proteins (e.g., ATIs), viral infections and gut microbiota, which are included in the list of candidate agents potentially contributing to the determination of CD risk in genetically predisposed individuals exposed to dietary gluten intake.

## Figures and Tables

**Table 1:** Overview and general characteristics of human toll-like receptors (TLRs).

TLRs	TLR Location	Adaptor Protein	Kinases	Transcription Factors	Induced Cytokines	Ligand	Potential Pathogen Target
TLR1	Cell membrane	MyD88 TIRAP	IRAK TAK1 IKK complex	NF-κB AP-1 IRF1 IRF5 IRF7	IL-6 IL-8 IL-10 IL-12 TNF-α	Triacyl-lipopeptide	bacteria mycobacteria
TLR2	Cell membrane	MyD88 TIRAP	IRAK TAK1 IKK complex	IRF3 IRF7 AP1	IFN-α IFN-β IFN-γ TNF-α IL-1 β IL-6 IL-8 IL-10 IL-12	Peptidoglycan	Gram+ bacteria
						Lipotechoic acid	Gram+ bacteria
						GPI-linked proteins	Trypanosoma *spp.*
						Phospholipomannan	*Candida albicans*
						Zymosan	*S. cerevisiae*
						Lipoprotein	bacteria
						Atypical LPS	Leptospira *spp.*
						Lipoarabinomannan	Mycobacteria
						Glycolipids	*T. maltophilum*
						Phenol-soluble modulin	*S. aureus*
						Glycoinositol-phospholipids	*T. cruzi*
						LPG	*Leishmania major*
						Galbeta1,4Manalpha-Po(4)- containing phospho-glycans	*Leishmania donovani*
						GPI anchor	*P. falciparum*
						Native GPI anchors	*T. gondii*
						Non-GPI anchors	*T. cruzi*
TLR3	Endosome	TRIF TRAF3	TAK1 IKK complex TBK1	NF-kB IRF3	IFN-α IFN-β TNF-α IL-12p40 IFN- γ	dsRNA	viruses
TLR4	Cell membrane; (Endosome)	MyD88 TIRA pTRAM TRIF	TAK1 TBK1	NF-kB IRF3 AP1	IFN-α IFN-β TNF-α IFN-γ IL-1 IL-2 IL-6 IL-10 IL-12	LPS	Gram- bacteria
						Mannan	*S.cerevisiae,C.albicasns*
						Glucuronoxylomannan	*C. neoformans*
						Fusion protein	RSV
						EDA domain fibronectin	(endogenous)
						HSP60	(endogenous)
						Glycoinositol-phospholipids	*T. cruzi*
						GPI anchor	*P. falciparum*
						Native GPI anchors	*T. gondii*
						Taxol	(synthetic)
						HSP70	(endogenous)
						Hyaluronum	(endogenous)
TLR5	Cell membrane	MyD88 TRAF6	IRAK-4 TAK1 IKK complex	NF-kB AP1	TNF-α IL-12	Flagellin	Flagellated bacteria
TLR6	Cell membrane	MyD88 TIRAP	IRAK TAK1 IKK complex	NF-κB AP-1 IRF1 IRF5 IRF7	TNF-α IL-6 IL-8 IL-10 IL-12	Diacyl-lipopeptide	Mycoplasma spp.
						Lipoteichoic acid	Gram+ bacteria
						GPI anchor	*T. cruzi*
						Zymosan	*S. cerevisiae*
TLR7	Cell membrane; Endosome	MyD88 TRAF6 TRAF3	IRAK1 IKKa	IRF7 AP1	IFN-α IFN-β	ssRNA	viruses
		MyD88 TRAF6	IKK complex	NF-kB AP1	TNF-α IL-6 IL-12	Imiquimod, R848	(synthetic)
						Loxiribine	(synthetic)
TLR8	Endosome	MyD88	IRAK	NF-κB AP-1 IRF1 IRF5 IRF7	IFN-α IFN-β	ssRNA	viruses
						R848	(synthetic)
TLR9	Endosome	MyD88 TRAF6 TRAF3	IRAK1 IKKa	IRF7 AP1	IFN-α IFN-β	Unmethylated CpG DNA	bacteria
						Herpes virus DNA	viruses
						CpG DNA	bacteria & viruses
						CpG ODNs	(synthetic)
		MyD88 TRAF6	IKK complex	NF-kB AP1	TNF-α IL-6 IL-12	Genomic DNA	*B. bovis, T. cruzi, * *T. brucei*
						Hemozoin	*P. falciparum*
						Unmethylated CpGDNA	bacteria
						Herpes virus DNA virus	viruses
TLR10	Cell membrane; (Endosome)	MyD88	IRAK?	?	?	HIV-1 gp41	viruses
						glycoprotein	viruses
						dsDNA	viruses

**Abbreviations**: TLR, toll-like receptor; MyD88, myeloid differentiation factor-88; TIRAP, toll-IL-1 receptor (TIR)-domain-containing adaptor protein; TRIF, toll receptor-associated activator of interferon; TRAF, tumor necrosis factor receptor-associated factor; TIRAP, TIR domain containing adaptor protein; TRAM, toll receptor-associated molecule; IL, interleukin; IRAK, IL-1 receptor-associated kinase; TAK1, transforming growth factor-β (TGF-β)-activated kinase 1; IKK complex, kinase complex made of two kinases (IKKα and IKKβ) and a regulatory subunit, NEMO/IKK γ; NF-κB, nuclear factor kappa-light-chain-enhancer of activated B cells; AP-1, activator protein; IRF, interferon regulatory factor; IFN, interferon; TNF, tumor necrosis factor; T. cruzi, trypanosome cruzi; Staph. Aureus, staphylococcus aureus; Atypical LPS, atypical lipopolysaccharide; Tc52, T. cruzi-released protein related to thiol-disulfide oxidoreductase family, called Tc52; GPI-linked proteins, glycosyl phosphatidylinositol -linked proteins; Non-GPI anchors, non-glycosyl phosphatidylinositol anchors; dsRNA, double-stranded RNA; LPS, lipopolysaccharide; EDA domain fibronectin, (endogenous)-fibronectin extra domain A; HSP 60, heat shock protein 60; HSP70, heat shock protein 70; ssRNA, single-stranded RNA; T. brucei, trypanosome brucei; P. falciparum, Plasmodium falciparum; RSV, respiratory syncytial virus; R848 (synthetic), resiquimod; DNA, deoxyribonucleic acid; Unmethylated CpG DNA, unmethylated cytosine–guanine dinucleotide DNA; CpG ODNs (synthetic), cytosine–guanine dinucleotide containing single-stranded DNA molecules containing cytosine–guanine dinucleotide motifs; HIV-1 gp41, glycoprotein 41 of human immunodeficiency virus.

**Table 2:** Ex vivo/in vitro cellular experiments investigating TLRs in the response of innate immune cells to gluten components.

Author (Year) [Ref.]	Study Objectives	Cellular System	Cell Source	Cell Stimulation	Relevant Methods	TLRs-Related Findings
Nikulina, et al. (2004) [39]	“to study whether wheat gluten exerts any specific effect on DCs maturation or function”	Bone marrow- derived dendritic cells (BMDC)	- C3H/HeJ * - C3H/HeN *	- chymotrypsin-digested wheat gluten - LPS	- FACS (MHC class II, CD40, CD54, CD86) - ELISA (TNF-ɑ, IL-10, MCP-1, IL-1β, MIP-2, MIP-1ɑ, and keratinocyte-derived cytokine)	- “TLR4 is not involved in gluten-induced BMDC maturation.”
Thomas, et al. (2006) [40]	“to test the hypothesis that gliadin initiates CD by stimulating the innate immune response”	Peritoneal macrophages + CHO reporter cell lines (3E10 & 3E10-huTLR2) *	Macrophages: - C57BL/6J - C3H/OuJ * - C3H/HeJ * - TLR4^−^/^−^ - -MyD88^−^/^−^	- Pepsin/trypsin-digested gliadin - LPS	- ELISA (TNF-ɑ, IL-12 p40) - mRNA expression analysis (TNF-ɑ, IL-12 p35, IL-12 p40, IL-15, IL-6, IFN-ɣ, iNOS, MCP-5, TLR2, TLR4)	- “Gliadin-induced zonulin release, increased intestinal permeability, and cytokine production were dependent on myeloid differentiation factor 88, a key adapter molecule in the TLR/IL-1R signalling pathways, but were neither TLR2- nor TLR4-dependent.”
Ciccocioppo, et al. (2008) [41]	“to study the immunological effects of gliadin stimulation on DCs from HLA-DQ8 transgenic and BALB/c mice”	Bone marrow dendritic cells	- DQ8/Ab^0^ * - BALB/c	- ɑ-chymotrypsin-digested gliadin - (ct-gliadin) - ovoalbumin	- FACS (HLA-DQ8, MHC class II, CD80, CD86, CD11c, CD3, CD4) - ELISA (IL-4, IL-10, IL-12p70, IFN-ɑ) - mRNA expression analysis (TLR4, TLR7, TLR8, TLR9)	- “Our findings showed that ct-gliadin caused a significant upregulation of TLR4, TLR7 and TLR8 mRNA levels in DQ8 DCs, while only TLR8 mRNA level was increased in BALB/c DCs.”
Palova-Jelınkova, et al. (2013) [42]	To analyse “the production of IL-1 cytokine family members…after stimulation with peptic digest of wheat gliadin fraction”	Mouse bone-marrow dendritic cells (BMDCs) + Human (CD patients) peripheral blood cells (PBMCs)	Mouse BMDCs: - C57BL/6 - Nlrp3^−^/^−^ - ASC^−^/^−^ - IL-1R^−^/^−^ - MyD88^−^/^−^ - TRIF^−^/^−^ - TLR4^−^/^−^ - TLR2^−^/^−^ - TLR2-4^−^/^−^	- PDWGF - ATP - Z-YVAD-fmk - LPS - Ovoalbumin	- ELISA (IL-1 ɑ, IL-18, IL-1β, TNF-ɑ) - FACS (CD40, CD80, CD86) - Western blotting (pro-IL-1β, IL-1β)	- “the secretion of IL-1β induced by PDWGF alone or in combination with ATP, was significantly reduced in MyD88 and TRIF-deficient BMDC and […] in TLR4 KO BMDC and abrogated in BMDC from mice deficient for both TLR2 and TLR4.”
Herrera, et al. (2018) [43]	“to investigate the role of 33-mer gliadin peptides in the activation of TLRs in macrophages.”	Bone marrow-derived macrophages (BMDMs) + HEK 293 Cells* + RAW-Blue™ Cells*	BMDMs: - C57BL/6 - TLR4^−^/^−^ - TLR2^−^/^−^	- 33-mer gliadin peptide - LPS - Pam3CSK4	- ELISA (TNF-ɑ, IP-10/CXCL10)	- “33-mer supramolecular structures larger than 220 nm are responsible for NF-κB activation and pro-inflammatory cytokine secretion through TLR4 and TLR2 activation.”
Nanayakkara, et al. (2018) [44]	To test “whether the A-gliadin peptide P31–43 could mimic and reinforce the IFN-α mediated innate immune response to viruses”	CaCo-2 Cells *	---	- P31–43 gliadin peptide - LOX (loxoribine—TLR7-specific ligand)	- Western blotting (MxA, ERK1/2, IFN-α, TLR7, MyD88, pY-NF-κB, NF-κB, pY-p38, p38, pY-JNK, JNK, HRS) - Immunoprecipitation (TLR-7, MyD88, EGFR) - mRNA expression analysis (IFN-α, MxA, TLR7, MyD88)	- “A-gliadin P31–43 activated IFN-α, a mediator of the innate immune response in CD, in the intestine of subjects with CD [see text] and an enterocyte cell line, CaCo-2. P31–43 cooperated with a viral ligand to activate the TLR7 pathway by interfering with endocytic trafficking.”

**Abbreviations**: “−/−”, knock-out mouse; Ab, antibody; ASC, apoptosis-associated speck like protein; ATP, adenosine triphosphate; BMDCs, bone marrow-derived dendritic cells; BMDMs, bone marrow-derived macrophages; CD, cluster of differentiation; CHO, Chinese hamster ovary; ct-gliadin, ɑ-chymotrypsin-digested gliadin; CXCL10, C-X-C motif chemokine ligand 10; DC, dendritic cell; EGFR, epidermal growth factor receptor; ELISA, enzyme-linked immunosorbent assay; ERK, extracellular signal-regulated kinase; FACS, fluorescence activated cell sorting; HEK, human embryonic kidney; HLA, human leukocyte antigen; HRS, hepatocyte growth factor-regulated tyrosine kinase substrate; IFN, interferon; IL, interleukin; IL-1R, interleukin-1 receptor; iNOS, inducible nitric oxide synthase; IP-10, interferon-gamma-inducible protein 10; JNK, c-Jun N-terminal kinase; LOX, loxoribine; LPS, lipopolysaccharide; MCP, murine monocyte chemoattractant protein; MD2, myeloid differentiation factor 2; MHC, major histocompatibility complex; MIP, macrophage inflammatory protein; MxA, myxovirus resistance protein; MyD, myeloid differentiation primary response; NF-κB, nuclear factor-κB; Nlrp, Nod-like receptor family containing pyrin domain; PAM3CK4, Pam3CysSerLys4 (TLR2 ligand); PBMC, peripheral blood mononuclear cells; PDWGF, peptic digest of wheat gliadin fraction; pY, phosphorylation; SEAP, secreted embryonic alkaline phosphatase; TLR, toll-like receptor; TNF, tumor necrosis factor; TRIF, TIR-domain-containing adapter-inducing interferon-β; Z-YVAD-fmk, benzyloxycarbonyl-Tyr-Val-Ala-Asp(OMe)-fluoromethylketone (caspase-1 inhibitor); PDWGF, peptic digest of wheat gliadin fraction. * CHO, Chinese hamster ovary; 3E10, CHO reporter cell lines expressing endogenous hamster TLR4 responds to TLR4 agonists, but not to TLR2 agonists; 3E10-huTLR2, CHO reporter cell line also expressing human TLR2 and, thus, responds to both TLR4 and TLR2 agonists; RAW-Blue™ Cells, murine RAW 264.7 macrophages with chromosomal integration of a secreted embryonic alkaline phosphatase (SEAP) reporter construct inducible by NF-κB and AP-1; HEK293 cells, human embryonic kidney cell line 293 (transfected with human TLR4 or human TLR2); CaCo-2 cells, immortalized cell line of human colorectal adenocarcinoma cells; C3H/HeJ, LPS hypo-responsive TLR4-mutated; C3H/HeN, wild-type TLR4; C3H/OuJ, homozygous for the retinal degeneration 1 mutation; -DQ8/Ab^0^, transgenic mice expressing the human HLA-DQ8 molecule in the absence of endogenous MHC class II genes.

**Table 3:** Animal studies assessing TLRs in the response of innate cells to gluten components.

Authors (Year) [Ref.]	Study Objectives	Murine Model	Experimental Conditions	Relevant Methods	General Findings	TLRs-Related Findings
Silva, et al. (2012) [51]	To investigate “whether treatment with gliadin induces paracellular permeability defect that enhances bacterial translocation to mesenteric lymph nodes via resident dendritic cells expressing TLR-2 or 4”.	- HCD4/HLA DQ8 * - C57BL/6	- sensitization: intraperitoneal injection of gliadin. - challenge (1 week later): gliadin by intragastric gavage (three times a week, for 7 weeks)	- Immunostaining (CD3, CD103, CD11c, TLR2, TLR4) - Tissue culture to assess the bacterial translocation - Analysis of intestinal paracellular permeability by ^51^Cr-EDTA	- “gliadin sensitization and oral challenge in HCD4/HLA-DQ8 transgenic mice is associated with increased para-cellular permeability and bacterial translocation to mesenteric lymph nodes and augmented number of transepithelial CD103+ cells”	- “…the effect of gliadin is TLR-2 and 4 independent. Thus, TLR-2 and 4 do not seem to play a role in this model of gliadin sensitivity, although the role of - other pattern recognition receptors cannot be ruled out”.
Araya, et al. (2014) [52]	To investigate “the inflammatory pathways elicited after intraluminal administration of poly I:C and determine acute and delayed consequences of this locally induced immune activation”.	- NODAB^0^DQ8 * - C57BL/6J	- C57BL/6J mice: - GFD - Intraluminal administration of poly I:C or PBS 3h, 12h - NOD-DQ8 mice: - Dietary antigen: gavage 5 mg of α-gliadin/time by oral gavage, three times a week, for 2 weeks - Mice weight three times a week during the 2 weeks.	- mRNA expression analysis (IFN-β, CXCL-10,TNFα, TLR3) - FACS (TNFα, MCP-1, IL-6, IL-10, IL-12p70) - assessment of intestinal barrier function by Ussing chambers	- “The severe enteropathy induced by intraluminal poly I:C, predisposed to functional impairment of small intestine in NODDQ8 mice receiving the dietary antigen, gliadin”	- “Inflammatory mediators and receptors MDA5, RIGI and TLR3 are induced by intraluminal poly I:C in small intestine”.
Marafini, et al. (2015) [53]	“to characterize the tissue distribution of ILCs in celiac disease (CD), […] and analyze their role in gut tissue damage”	- RAG1−/−	RAG1−/− mice: - injection with phosphate buffered saline (CTR) and poly I:C	- FACS (CD56, CD45, IFN-γ, TNF-α, TLR3, TLR9, TLR4, TLR2, ROR-γt, ROR-γt, T-bet, IL-17A, TLR7)	- “CD-related inflammation is marked by accumulation of ILCs producing TNF-α and IFN-γ in the mucosa.”	- “ILCs expressed TLR2, TLR3 and TLR9 but neither TLR7 nor TLR4. […] ILCs express TLR3 and are functionally able to respond to poly I:C with increased synthesis of TNF-α thus contributing to small intestinal atrophy”.
Araya, et al. (2016) [54]	To determine “whether p31–43 elicits innate immune activation in murine small intestine in vivo and to investigate potential underlying pathways.”	- MyD88−/− - IFNR−/− - C3H-HeJ * - C57BL/6	- Intraluminal administration of p31–43 peptide, Poly I:C, p31–43+Poly I:C	- mRNA expression analysis (IFN-γ, IFN-β, CXCL10, CXCL2, IL-15, IL-18, IL-1, IL-6, TNFα-, MCP1, CXCR3, Bax and Bcl2) - Confocal microscopy with TUNEL reaction	- “p31–43 caused an inflammatory response in the small intestine, characterized by elevation of IFN-γ expression followed by elevations in CXCL10. p31–43 also induced cell death in epithelial cells. Finally, we demonstrated that the mucosal damage induced by p31–43 is type I IFN dependent.”	- “A direct proinflammatory effect of p31–43 in vivo that requires the central adaptor of the TLR pathway, MyD88, but is independent of TLR4”.

**Abbreviations**: “−/−”, knock-out mouse; TLR, toll-like receptor; ^51^Cr-EDTA, chromium-ethylenediaminetetraacetic acid; HLA, human leucocyte antigen; CD3, cluster of differentiation 3; GFD, gluten-free diet; Poly I:C, polyinosinic:polycytidylic acid; PBS, phosphate-buffered saline; RTPCR, real time- polymerase chain reaction; IFN, interferon; MDA5, melanoma differentiation-associated gene 5; RIGI, retinoic acid-inducible gene I; CXCL10, C-X-C motif ligand 10; CXCR3, C-X-C motif chemokine receptor 3; TNFα, tumor necrosis factor Alpha; Bcl2, B-cell lymphoma 2 protein; Bax, B-cell lymphoma associated X protein; MCP-1, monocyte chemoattractant protein 1; IL, interleukin; CTR, healthy controls; FACS, fluorescence-activated single cell sorting; RORγt, retinoic acid receptor-related orphan receptor gamma; T-bet, T-box transcription factor; ILCs, innate lymphoid cells; TNFα tumor necrosis Alfa; RAG1, recombinase-activating gene1; MyD88−/−, myeloid differentiation factor-88 knockout mice; IFNR−/−, interferon knockout mice; p31–43, α-gliadin peptide P31–43. * HCD4/HLA-DQ8, transgenic mouse expressing HLA-DQ8 genes in the absence of endogenous mouse MHC class II genes; NOD AB^0^ DQ8, cross from trans-genic HLA DQ2/DQ8 and MHCII-deficient mouse with NOD mouse; C3H/HeJ, LPS hypo-responsive TLR4-mutated.

**Table 4:** Clinical studies assessing TLRs in patients affected with Celiac Disease.

Authors (Year) [Ref.]	CD Patients (N) [M:F; µ/m,R]	Other Groups (N) [M:F; µ/m,R]	TLRs Focus	Relevant Methods	Sample	General Findings	TLRs-Related Findings in CD
Zanoni, et al. (2006) [55]	***gfdCD*** (*n* = 22) [4:18; µ:16 years, 1–38 years] ***aCD*** (*n* = 38) [12:26; µ:14 years, 1–56 years]	***Healthy*** ***individuals*** (*n* = 60) [“age-/sex-matched”] ***Other autoimmune diseases*** (*n* = 180) [n/a]	TLR4	- CD autoantibodies by DELFIA and ELISA - Monocytes FACS analysis (CD1, CD83, CD86, CD40) - Cytokines Measurement by ELISA (IL-6, IL-12, TNF-α)	Peripheral blood	- “…in active CD, autoantibodies against an epitope of tTG bind other self-antigens and the rotavirus-derived protein VP-7, […] Moreover, such self-reactive antibodies are pathogenetically relevant for their ability to alter the intestinal barrier integrity and to activate monocytes.”	- “purified anti-rotavirus VP-7 peptide antibodies are able to cross-react with the celiac peptide, desmoglein peptide, and TLR4 peptide.” - “The anti-celiac peptide antibodies bind TLR4 on monocytes and induce both the expression of activation molecules such as CD83 and CD40 and the production of pro-inflammatory cytokines in an extent similar to LPS.
Szebeni, et al. (2007) [56]	***gfdCD*** (*n* = 9) [4:5; m:6 years, 3–14 years] ***aCD*** (*n* = 16) [6:10; m:9 years, 4–15 years]	***“children […] who were investigated for either growth*** ***retardation*** ***or chronic diarrhea”*** (*n* = 10) [4:6; m:10 years, 4–15 years]	TLR2 TLR3 TLR4	- mRNA expression analysis by RT-PCR (TLR2, TLR3, TLR4) - Protein expression analysis by Western blotting (TLR2, TLR3, TLR4)	Duodenal mucosa	---	- “…significant upregulation of TLR2 and TLR4 mRNA expression in the duodenal mucosa of children with untreated CD and treated CD compared with controls. - “…TLR2 and TLR4 mRNA levels were even higher in the duodenal mucosa of children with treated CD compared with untreated CD.” - “…increased TLR2 and TLR4 protein levels in children with untreated CD and treated CD. Moreover, TLR2 and TLR4 protein levels were even higher in the duodenal mucosa of children with treated CD than in those with untreated CD.” - “TLR3 mRNA expression was increased in the duodenal mucosa of children with treated CD compared with untreated CD and controls. We were able to detect TLR3 protein only in the biopsy specimens of patients with treated CD.”
Santin, et al. (2007) [57]	***CD*** (*n* = 281) [*n*/a]	***“Healthy Unrelated Controls”*** (*n* = 186) [n/a]	TLR4	- PCR-based TLR4 SNPs analysis (Asp299Gly and Thr399Ile)	Peripheral Blood	---	- “Our results do not support association of the two TLR4 SNP variants (Asp299Gly and Thr399Ile) with CD.”
Deszofi, et al. (2008) [58]	***CD*** (*n* = 100) [47:53; m:16 years, 3–40 years] ***CD + T1DM*** (*n* = 47) [21:26; m:14 years, 3–20 years]	***T1DM*** (*n* = 80) [41:39; m:15 years, 4–22 years]	TLR4	- Genotyping by PCR-RFLP (CD14 C^−260T^, TLR4 A^+896^G)	Peripheral Blood	- “… in patients with T1DM, the CD14 C-260TT homozygous genotype increases the risk for the development of CD.”	- “No difference was found in the genotype and allele frequencies of TLR4 between the studied groups.”
Cseh, et al. (2010) [59]	***aCD*** and ***gfdCD*** (*n* = 10) [4:6; m:3 years, 2–5 years]	***“children with functional*** ***non-organic abdominal pain”*** (*n* = 15) [6:9; [m:3 years, 2–6 years]	TLR2 TLR4	- PBMC analysis by FACS (CD3, CD4, CD8, CD11c, CD14, CD25, CD45RA, CD45RO, CD62L, CD69, CD123, CD161, CXCR3, HLA-DR, Lin-1, TLR2, TLR4, FoxP3)	Peripheral Blood	- “…immune phenotype in childhood CD exhibits several abnormalities: these include the increased activation of both adaptive and innate immunity.” - “The alterations of adaptive immunity are mainly normalized with GFD, while those of innate immunity still persist in spite of the clinical improvement of CD-associated signs and symptoms.”	- “APCs expressing TLR-2 and TLR-4 receptors were more prevalent in CD.” - “The prevalence of APCs expressing TLR-4 normalized after introduction of GFD in CD patients.” - “After resolution of symptoms on GFD…the prevalence of TLR-2 expressing DCs and monocytes remained abnormal.”
Fernandez-Jimenez, et al. (2010) [60]	***CD*** (*n* = 376) [135:241; µ:3.8 years, n/a]	***“Adult controls”*** (*n* = 376) [154:222; n/a]	TLR2 TLR4	- Copy number variation analyses by RT-PCR (TLR2, TLR4, β-defensin gene cluster)	Peripheral Blood	- “β-defensin clusters varied between 2 and 9 copies per genome […] high copy numbers were under-represented among patients”	- “TLR genes did not show copy number variation, and all samples presented with two copies.”
Westerholm- Ormio, et al. (2010) [61]	***aCD*** (*n* = 10) [4:6; µ:4.4 years, 1.9–6.7 years]	***“Healthy controls”*** (*n* = 10) [6:4; µ:4 years, 1.1–6.7 years] ***Untreated food allergy*** (*n* = 7) [4:3; [µ:2.1 years, 0.6–5.4 years] ***Treated food allergy*** (*n* = 12) [7:5; [µ:4 years, 0.3–6.9 years]	TLR2 TLR4	- Immunostaining (CD3, CD4, CD8, CD25, ɣ𝛿TCR, CTLA-4, Foxp3, IL-4, IL-10 IFN-ɣ, TGF-β1, TLR2, TLR4) - Light Microscopy - mRNA expression analysis by RT-PCR (CD25, TLR2, TLR4, Foxp3)	Duodenal Mucosa	- “The density of CD25+ and CTLA-4+ cells were significantly higher in patients with CD than in the other study group.“ - “Patients with CD had significantly higher densities of CD4+Foxp3+-, CD25+Foxp3+-, and CTLA-4+Foxp3+-expressing cells than healthy controls did.” - “The expression of Foxp3 mRNA was significantly higher in the small intestinal mucosa of patients with CD than in untreated patients with FA. The expression of CD25 mRNA did not differ significantly between the study groups”.	- “The density of TLR4+ cells in the lamina propria was higher in untreated patients with FA than in control patients. Patients with CD also showed higher density of TLR4^+^ cells than control patients did.” - “The expression of TLR2 mRNA was significantly increased in the small intestinal mucosa of control patients compared with patients with CD. The expression of TLR4 mRNA did not differ significantly between the study groups”.
Kalliomaki, et al. (2011) [62]	***aCD*** (*n* = 10) [n/a; µ:9.5 years, 3–14 years] ***gfdCD*** (*n* = 6) [n/a; µ:46 years, 30–60 years]	***“control children”*** (*n* = 9) [n/a; µ:8.5 years, 4–16 years]	TLR2 TLR3 TLR4 TLR5 TLR9	- mRNA expression analysis by RT-PCR (IL-8, TLR2, TLR3, TLR4, TLR5, TLR9, TOLLIP) - Gut microbiota analysis by qPCR and SYBR Green chemistry	Duodenal Mucosa	- “Small intestinal microbiota was comparable among controls, untreated celiacs, and treated celiacs. Expression of IL-8 mRNA, a marker of intestinal inflammation, was significantly increased in untreated celiacs as compared with treated celiacs (*p* = 0.002) and controls (*p* = 0.001).”	- “Expression of TLR-2 mRNA was significantly decreased in untreated (*p* = 0.001) and treated (*p* = 0.03) celiacs, whereas expression of TLR-9 mRNA was increased in untreated celiacs (*p* = 0.001) as compared with controls. Expression of TOLLIP mRNA was downregulated in untreated.”
Eiro, et al. (2012) [63]	***CD children*** (*n* = 10) [5:5; m:7.5 years, <14 years] ***CD*** ***adults*** (*n* = 31) [7:24; m:42.5 years, n/a]	***“patients with pain in the epigastrium*** ***without CD or*** ***H. pylori infection”*** (*n* = 21) [9:12] - Children (*n* = 10) [m:7 years, n/a] - Adults (*n* = 11) [m:50.6 years, n/a]	TLR3 TLR4 TLR7	- Immunostaining (TLR3, TLR4, TLR7) - mRNA expression analysis by RT-PCR (TLR3, TLR4, TLR7, IL1, IL6, IL8, IL17, IRAK4, MyD88, NF-κB)	Duodenal Mucosa	- “This study demonstrated a similar inflammatory profile in children and in adult disease,…”.	- “…samples obtained from adults and children with CD were compared, and the results showed no significant differences for TLR3, TLR4, and TLR7 expression levels between these groups.” - “TLR4 expression level was increased twofold in CD specimens compared to controls.”
Cheng, et al. (2013) [64]	***children*** ***aCD*** (*n* = 10). [4:6; m:9.5 years, 3–14 years] ***adults*** ***gfdCD*** (*n* = 6) [n/a; m:46 years, 30–60 years]	***“Healthy*** ***Control children with gastro-intestinal*** ***complaints”*** (*n* = 10) [4:6; m:8.5 years, 4–16 years]	TLR2 TLR9	- RT-PCR (IL-10, IFN-ɣ, TNF-α, ZO-1, CX43, MUC2, RegIIIγ, CXCL16, CXCR6) - Microbiota analysis by phylogenetic microarray and relative content estimation	Duodenal Mucosa	- “The overall composition, diversity and the estimated microbe associated molecular pattern (MAMP) content of microbiota were comparable between CD and HC, but a sub-population profile comprising eight genus-like bacterial groups was found to differ significantly between HC and CD.”	- “In HC, increased TLR2 expression was positively correlated with the expression of tight junction protein ZO-1. In CD and T-CD, the expression of IL-10, IFN-ɣ and CXCR6 were higher as compared to HC.”
Marafini, et al. (2015) [53]	***aCD*** (*n* = 19) [n/a; m:33 years, 16–53 years] ***gfdCD*** (*n* = 14) [n/a; m:34 years, 18–50 years]	***“controls”*** (*n* = 17) [n/a; m:50 years, 30–87 years]	TLR2 TLR3 TLR4 TLR7 TLR9	- Flow cytometry (CD3, CD19, CD20,CD56, CD45, IFN-γ, TNF-α, TLR3, TLR9, TLR4, TLR2, ROR-γt, CD127, T-bet, IL-17A, TLR7, CD90.2, ROR-γt)	Duodenal Mucosa	- “CD-related inflammation is marked by accumulation of ILCs producing TNF-α and IFN-γ in the mucosa”	- “ILCs expressed TLR2, TLR3 and TLR9 but neither TLR7 nor TLR4”. - “ILCs express TLR3 and are functionally able to respond to poly I:C with increased synthesis of TNF-α thus contributing to small intestinal atrophy”
Brynychova, et al. (2016) [65]	***aCD*** (*n* = 10) ***gfdCD*** (*n* = 10). [0:20;m:30 years, n/a]	***“healthy individuals”*** (*n* = 10) [n/a; m:29.5 years; n/a]	TLR2 TLR4 TLR7	- RT-PCR (TNF-α, IL-6, IL-10, IL-12A, TLR2, TLR4, TLR7)	Peripheral blood	- “The data suggest that the inflammatory process in rCD intestinal mucosa and submucosa reflecting enterocyte damage can be detected in PBMs in peripheral blood”.	- “Further, the cytokine and TLR expression profile in PBMs alters after one year of GFD treatment” - “Increased levels of TLRs (TLR2, TLR4 and TLR7) are detectable in peripheral blood monocytes from patients with active celiac disease.”
Kumar, et al. (2017) [66]	***gfdCD*** (*n* = 30) [n/a]	***“healthy children”*** (*n* = 30) [n/a]	TLR2	- FACS (CD4, CD25, Foxp3, TLR2, IFNγ) - Biochemical assays to assess enzymatic and non-enzymatic parameters of oxidative stress	Peripheral blood	- “The present study is the first of its kind where we demonstrate the role of oxidative stress on peripheral regulatory cell population along with expression of Toll like receptor 2 (TLR2). By investigating oxidative stress in celiac disease, we could suggest its important role in CD”	- “Expression of TLR2 on iTregs was elevated in CD and GFD, however, expression on nTregs was unaltered in all the three groups”. - “A strong correlation was observed between the cytokine IFN-c and TLR2 expression in movement of iTregs in CD patients.”
Ghasiyari, et al. (2018) [67]	***CD*** (*n* = 120) [n/a; µ:34.5 years, n/a]	***“healthy individuals”*** (*n* = 120) [n/a; µ:40.4 years, n/a]	TLR2 TLR4 TLR7 TLR9	- PCR (TLR2, TLR4, TLR7, TLR9, HLA DQ2/DQ8)	Peripheral blood Duodenal Mucosa	---	- “…statistically significant upregulation of TLR4 (*p* = 0.01) and TLR9 (*p* = 0.02) in blood samples of CD compared to HC but not for TLR2 (*p* = 0.13) and TLR7 (*p* = 0.08).” - “in duodenal mucosa of CD patients, mRNA expressions of TLR2 (*p* = 0.03) and TLR4 (*p* = 0. 003) were significantly increased, whereas TLR9 (*p* = 0.0001) expression was down-regulated in patients compared to controls.” - “no significant differences in the expression of TLR7 (*p* = 0.74) in biopsy specimens.”
Brynychova, et al. (2019) [68]	***aCD*** (*n* = 10) [2:8; µ:36.7 years, n/a]	***“healthy individuals”*** (*n* = 10) [2:8; [n/a; µ:37.1 years, n/a]	TLR9	- Isolation and quantification of cfDNA by qPCR - TLR9 mRNA expression analysis in monocytic cell line THP1 upon stimulations (including plasma from healthy controls and CD patients)	Peripheral blood	- “… Despite the fact that celiac disease is autoimmune and inflammatory disease, we did not observe higher levels of cfDNA in patients’ plasma in comparison with healthy ageand sex-matched controls.”	- “ We documented the ability of cfDNA contained in CD plasma samples to stimulate the production of TLR9 mRNA. The TLR9 mRNA levels were significantly (*p* = 0.014) lower after cfDNA removal from CD plasma samples. No differences were observed in experiments performed with HC plasma samples.
Cerqueira, et al. (2021) [69]	***CD*** (*n* = 625) [n/a; m:41 years, 1–79 years]	***“healthy controls”*** (*n* = 1817) [n/a]	TLR7 TLR8	- HLA-DQ genotyping - Polygenic risk score (PRS) based on genome-wide association study (GWAS)	Peripheral blood	- “we identified a genotype-phenotype association for ten SNPs previously associated with coeliac disease susceptibility. - “combining 39 coeliac disease-associated genotyped SNPs into wGRS39 was more informative than a PRS to assess the genetic risk for distinct phenotypes of coeliac disease.”	- “According to our results, rs5979785 located in the proximity of the TLR7 and TLR8 genes was associated with a decreased risk of diagnosis before 7 years of age in girls and notably this association was detected also in the case only comparison. Our RegulomeDB analysis did not retrieve rs5979785 as having regulatory function of transcription…”

**Abbreviations**: CD, celiac disease; Ref., reference number; N or n, patients’ number; M:F, male to female ratio; µ, mean; m, median; R, age range; GFD, gluten-free diet; aCD, active CD; gdfCD, CD on gluten free diet; n/a, information not available; SNP(s), single nucleotide polymorphism(s); FACS, fluorescence-activated cell sorting; PCR, polymerase chain reaction; RT-PCR, real-time PCR; DELFIA, dissociation-enhanced lanthanide fluorescence immunoassay; ELISA, enzyme-linked immunoassay; cfDNA, cell-free DNA.

## Data Availability

Not applicable.
